# Neutrophil-associated plasma proteomics identifies HDAC1 as a baseline biomarker of immune tolerance during immunosuppressant withdrawal after pediatric liver transplantation: a single-center cohort study

**DOI:** 10.3389/fimmu.2026.1800926

**Published:** 2026-03-26

**Authors:** Ruofan Wang, Tianran Chen, Yifan Liu, Weiping Zheng, Chong Dong, Kai Wang, Chao Sun, Jing Chen, Enbo Xie, Yang Yang, Zhen Wang, Chao Han, Zhixin Zhang, Shengqiao Zhao, Xinzhe Wei, Guoyin Zou, Feiruzha Fulati, Zhuolun Song, Wei Gao

**Affiliations:** 1 First Central Hospital of Tianjin Medical University, Tianjin, China; 2 Department of Hepatobiliary and Pancreatic Surgery, The First Affiliated Hospital of Zhengzhou University, Zhengzhou, China; 3 Department of Liver Transplantation, Tianjin First Central Hospital, Tianjin, China; 4 School of Medicine, Nankai University, Tianjin, China; 5 Tianjin Key Laboratory of Organ Transplantation, Tianjin First Central Hospital, Tianjin, China

**Keywords:** HDAC1, immune tolerance, immunosuppressant withdrawal, neutrophils, pediatric liver transplantation, plasma proteomics

## Abstract

**Background:**

In pediatric liver transplantation (pLT), long-term immunosuppression (IS) contributes to infection risk and chronic toxicity, yet IS minimization or withdrawal requires balancing rejection risk. Practical biomarkers for baseline risk stratification at the start of planned withdrawal remain scarce. This study investigated whether baseline neutrophil-associated proteomic signatures and histone deacetylase 1 (HDAC1) levels are associated with IS withdrawal outcomes.

**Methods:**

Within an institutional IS withdrawal program (n = 77), 59 recipients had evaluable outcomes by the follow-up cut-off (June 30, 2025). Among these, 31 pLT recipients underwent planned IS withdrawal (primary analytic cohort), and baseline plasma from 10 patients was analyzed via liquid chromatography-mass spectrometry (LC-MS) proteomics to identify tolerance-associated proteins and pathways. HDAC1 was subsequently quantified by ELISA in 39 recipients with baseline plasma available. The diagnostic performance of HDAC1 in distinguishing immune-tolerant (IT) from non-immune-tolerant (NIT) outcomes was evaluated using receiver operating characteristic (ROC) analysis. To corroborate tissue-level consistency, HDAC1 expression was assessed by immunohistochemistry (IHC) in baseline liver biopsy sections from 10 recipients (5 IT and 5 NIT) selected from the planned withdrawal cohort.

**Results:**

Proteomic profiling revealed distinct baseline differences enriched in neutrophil-related functions, including pathways linked to degranulation and neutrophil extracellular trap (NET) formation. HDAC1 was identified as a key candidate marker, with significantly lower baseline levels observed in the IT group. In the validation cohort, plasma HDAC1 demonstrated moderate discriminative performance for baseline risk stratification (AUC = 0.81). Furthermore, IHC analysis of baseline liver biopsies showed lower intrahepatic HDAC1 staining in IT recipients compared to the NIT group, consistent with the systemic plasma findings.

**Conclusions:**

Baseline neutrophil-linked proteomic signals and diminished HDAC1 expression are associated with successful IS withdrawal in pLT recipients. These findings support HDAC1 as a hypothesis-generating candidate biomarker for baseline risk stratification and provide a clinically oriented framework to refine patient selection and enhance early monitoring during IS minimization and withdrawal protocols.

## 1 Introduction

Liver transplantation (LT) is the definitive life-saving intervention for children with end-stage liver disease; however, long-term graft and patient survival are as much a product of meticulous immunosuppression (IS) management as they are of surgical success. Achieving operational tolerance—defined as sustained graft stability in the absence of maintenance IS—remains a paramount clinical goal. This is particularly critical in the pediatric population, where recipients face decades of cumulative exposure to IS-related morbidities, including increased susceptibility to opportunistic infections, *de novo* malignancies, and progressive organ toxicity (1–3). Given that these risks are amplified over a child’s lifetime, planned immunosuppression tapering and withdrawal has emerged as a major focus of clinical research (4, 5). Nevertheless, the lack of robust, baseline biomarkers to identify suitable candidates for planned tapering and withdrawal remains a significant barrier to the widespread implementation of safe withdrawal strategies (6–8).

While clinical factors such as a younger age at transplant and a prolonged interval from transplantation to the start of planned immunosuppression tapering and withdrawal have been associated with higher tolerance rates, these metrics lack sufficient predictive precision (6, 7). Historically, the search for tolerance biomarkers has predominantly focused on adaptive immunity, particularly the role of T cell subpopulations and their associated transcriptional signatures (9–12). While this T cell-centric view is fundamental, it may not fully encompass the complex immune landscape encountered during planned immunosuppression tapering and withdrawal. Emerging evidence suggests that innate immune pathways significantly influence graft stability and the inflammatory milieu. Among these, neutrophils traditionally viewed as simple effector cells, are now recognized as sophisticated regulators of tissue inflammation and antimicrobial defense through processes such as degranulation and the formation of neutrophil extracellular traps (NETs). Recent studies have begun to link neutrophil-related pathways to transplant outcomes and the biology of tolerance in various organ contexts (13–15), yet their specific role in pediatric liver transplant (pLT) withdrawal remains poorly defined.

In this single-center study of pLT recipients undergoing planned immunosuppression withdrawal, we employed an unbiased plasma proteomic profiling approach to characterize the immune state at trial entry (baseline), prior to tapering. Our analysis identified neutrophil-associated pathways as the most prominent signatures distinguishing immune-tolerant (IT) from non-immune-tolerant (NIT) outcomes, with histone deacetylase 1 (HDAC1) emerging as a pivotal candidate marker. To validate these findings, we quantified baseline HDAC1 levels in a validation cohort via ELISA and evaluated its utility in clinical risk stratification. Furthermore, to bridge the gap between systemic signals and the local graft environment, we performed HDAC1 immunohistochemistry on baseline liver biopsies from a subset of recipients. Our results demonstrate that lower neutrophil-associated plasma signatures and lower HDAC1 expression are associated with successful withdrawal, providing a clinically oriented framework to refine patient selection and optimize IS minimization strategies.

## 2 Patients and methods

### 2.1 Study design and patient cohort

This single-center, retrospective cohort study included pediatric liver transplant recipients undergoing a structured immunosuppression (IS) minimization program with planned tacrolimus tapering and withdrawal between January 1, 2017, and June 30, 2025. In total, 77 recipients were enrolled in the withdrawal program. By the follow-up cut-off date (June 30, 2025), 18 recipients were still undergoing stepwise tapering or were in post-withdrawal follow-up without graft dysfunction and had not yet reached the predefined time point for outcome adjudication; therefore, 59 recipients were evaluable for outcome classification. Within the evaluable cohort, 31 recipients underwent planned/protocol-driven withdrawal (primary analytic cohort), whereas 28 underwent disease-/event-driven immunosuppression reduction (PTLD or other clinical indications). Biomarker analyses were performed using baseline samples collected at trial entry (before tapering): discovery proteomics was conducted in 10 recipients selected from the planned withdrawal cohort (n = 31), and ELISA validation was performed in 39 recipients with baseline plasma available. The overall cohort derivation and allocation to biomarker analyses are summarized in **Supplementary Figure 1**. Based on clinical outcomes and graft biopsy results following immunosuppressant reduction, patients were categorized into immune-tolerant (IT) and non-immune-tolerant (NIT) groups. The study was approved by the Ethics Committee of Tianjin First Central Hospital (2023DZX32), and written informed consent was obtained from parents or legal guardians of all participants. Clinical and demographic data were collected, including graft function, surgical details, immunosuppressive regimens, and blood samples obtained at trial entry (baseline) prior to IS withdrawal. All procedures adhered to the Declaration of Helsinki.

### 2.2 Criteria for patient enrollment and immunosuppressant withdrawal

Immunosuppressant withdrawal after pediatric liver transplantation can be classified as planned (protocol-driven) tapering and withdrawal, intended to achieve operational tolerance, or disease-driven immunosuppression reduction, prompted by complications such as post-transplant lymphoproliferative disorder (PTLD) or immunosuppression-related toxicity (e.g., renal impairment). Planned tacrolimus tapering and withdrawal in pediatric recipients followed the inclusion and exclusion criteria below.

Inclusion criteria.

1. Pediatric liver transplant recipients with age at transplantation < 6 years.

2. ≥ 4 years post-transplantation.

3. No rejection episodes and stable liver function during the 2 years before enrollment.

4. Tacrolimus (FK506) monotherapy.

5. No hepatitis B or hepatitis C infection within 1 year prior to enrollment.

6. Baseline liver function tests showing ALT, AST, and GGT all < 50 U/L.

Exclusion criteria.

1. Primary diseases including malignancy, autoimmune liver disease, viral hepatitis, repeat liver transplantation.

2. Any systemic condition requiring, or likely to require recurrent, immunosuppressive therapy.

3. Evidence of rejection or fibrosis on baseline liver biopsy (Ishak fibrosis score ≥ 2 or LAFSc >2).

**Figure 1:**
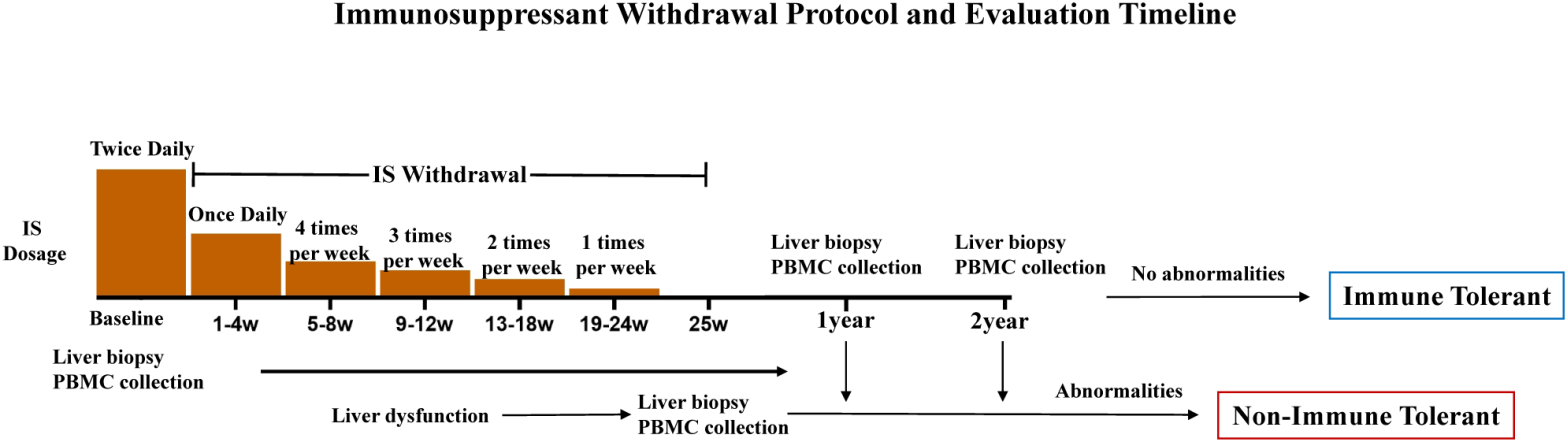
Immunosuppressant withdrawal protocol and evaluation timeline.

In our center, planned immunosuppression tapering and withdrawal in pediatric liver transplant recipients follows a stepwise tapering protocol, with progressive reductions in dosing frequency over six steps. The tapering period lasts approximately 25 weeks (**Figure 1**). Scheduled protocol liver biopsies are performed at 1 and 2 years after enrollment to assess graft histology. Immune tolerance is defined as stable liver biochemistry and absence of clinical or histological rejection at the 2-year post-enrollment assessment (including the scheduled year-2 protocol biopsy) after initiation of planned tapering/withdrawal. Withdrawal failure is defined by any of the following: liver function abnormality after enrollment that met the program criteria (if baseline values were within the normal range, ALT and/or AST > 2× the upper limit of normal; if baseline values were above normal, ALT and/or AST > 2× the baseline value, with or without concomitant elevation of ALP/GGT), confirmed on repeat testing, or biopsy evidence of rejection or fibrosis (Ishak fibrosis score ≥ 2 or LAFSc > 2). When biopsy was not obtained due to clinical constraints (e.g., contraindications or refusal), episodes meeting the above biochemical criteria with exclusion of alternative causes and improvement after prompt reinstitution/intensification of immunosuppression were adjudicated as clinical rejection and classified as withdrawal failure.

**Figure 2:**
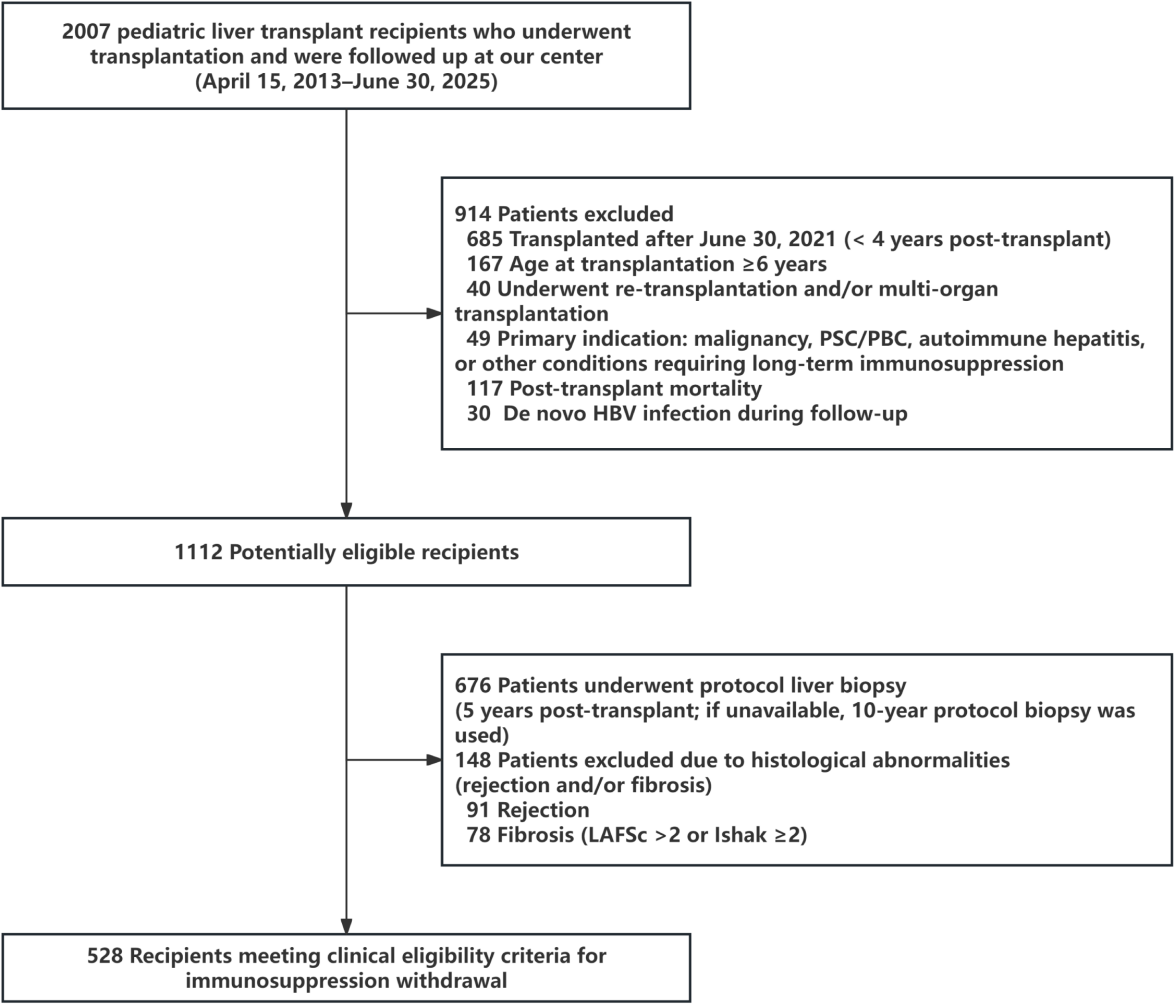
Flowchart for selection of potential candidates for immunosuppressant withdrawal in the pediatric liver transplant cohort.

To identify potential candidates for planned immunosuppression tapering and withdrawal, we screened 2,007 pediatric liver transplant recipients who underwent transplantation and were followed at our center between April 15, 2013 and June 30, 2025 (**Figure 2**). A total of 914 recipients were excluded due to one or more predefined conditions, including less than 4 years of follow-up by the cut-off date (i.e., transplantation after June 30, 2021; n = 685), age at transplantation ≥ 6 years (n = 167), repeat transplantation and/or multi-organ transplantation (n = 40), primary indication of malignancy, PSC/PBC, autoimmune hepatitis, or other conditions requiring long-term immunosuppression (n = 49), post-transplant mortality (n = 117), and *de novo* HBV infection during follow-up (n = 30). These exclusion categories were not mutually exclusive. After screening, 1,112 recipients remained potentially eligible. Among them, 676 underwent protocol liver biopsy at 5 years post-transplant; when a 5-year biopsy was unavailable, a 10-year protocol biopsy was used. Based on histological assessment, 148 recipients were excluded due to rejection and/or fibrosis, including rejection (n = 91) and fibrosis (LAFSc > 2 or Ishak ≥ 2; n = 78), with overlap between categories. Ultimately, 528 recipients met the clinical eligibility criteria for planned immunosuppression tapering and withdrawal.

### 2.3 Proteomic data analysis

Peripheral blood plasma samples were collected at trial entry (baseline) from 10 recipients undergoing planned tacrolimus withdrawal (IT, n = 5; NIT, n = 5) selected from the planned withdrawal cohort (n = 31) and subjected to proteomic profiling. Baseline characteristics of the proteomics cohort are shown in **Supplementary Table 1**. Proteomic data acquisition and processing were performed by Oebiotech Biological Technology Co., LTD (Qingdao, China) using a DIA workflow, and database searching/library generation were conducted in Spectronaut Pulsar (v18.4, Biognosys) with precursor and protein q-value cutoffs of 0.01 (1% FDR) and local normalization. Differential expression was assessed between IT and NIT, and differentially expressed proteins (DEPs) were defined as those with a fold change > 1.5 and a two-sided P value < 0.05. Benjamini–Hochberg FDR-adjusted P values were additionally reported to account for multiple testing, and proteins were filtered for quantification completeness prior to testing (no imputation). Functional annotation of identified proteins was performed using GO and KEGG databases, enrichment analyses were conducted for DEPs, and protein–protein interaction analysis was performed using the STRING database. Reactome pathway analysis and GSEA were performed using the full ranked protein list; key settings (background set, correction method, and GSEA parameters) are provided in the **Supplementary Materials**.

### 2.4 ELISA validation

To validate the findings from the proteomic analysis, ELISA was conducted on baseline plasma samples from a validation cohort consisting of 39 patients (IT: n = 17, NIT: n = 22). All samples were collected at trial entry (baseline), prior to immunosuppression tapering/withdrawal, and HDAC1 concentrations were measured and compared between the IT and NIT groups to evaluate their value for risk stratification of withdrawal outcomes. ELISA analysis was performed using the Human Histone Deacetylase 1 (HDAC1) ELISA kit (EK14440, SAB, USA). Assays and analyses were conducted according to the manufacturer’s protocol.

### 2.5 HDAC1 immunohistochemistry on FFPE liver biopsy

To corroborate the proteomic and ELISA findings at the tissue level, HDAC1 expression was evaluated by immunohistochemistry in baseline liver biopsy specimens. Baseline liver biopsies were performed at study entry in all 39 recipients in the validation cohort and archived as FFPE blocks. For tissue-level corroboration, HDAC1 IHC was performed in 10 planned-withdrawal recipients (5 IT and 5 NIT) selected based on proximity to the follow-up cut-off date (June 30, 2025) to minimize potential storage-related attenuation of antigen immunoreactivity. IHC staining was performed using a rabbit monoclonal anti-HDAC1 antibody (HUABIO, ET1605-35) at a dilution of 1:1000, following the manufacturer’s recommended protocol. Briefly, FFPE sections were deparaffinized and rehydrated through graded alcohols, followed by antigen retrieval. Endogenous peroxidase activity was quenched, and sections were incubated with the primary antibody. After washing, sections were incubated with an appropriate HRP-conjugated secondary antibody and visualized using a DAB chromogen system, with hematoxylin counterstaining. Stained slides were imaged under bright-field microscopy on a Nikon platform (scale bar, 50 μm) using identical acquisition settings across slides. Images were anonymized before analysis. Negative controls (omission of primary antibody) were included. Images were anonymized before analysis, and quantification was performed blinded to outcome group. HDAC1 immunoreactivity was quantified using ImageJ (NIH, USA; v1.54p) as the HDAC1-positive area fraction (DAB-positive area/total tissue area). For each case, five non-overlapping parenchymal fields were analyzed, and a fixed threshold was applied uniformly to identify DAB-positive signals; the mean value across fields was used for comparison between IT and NIT groups.

### 2.6 Statistical analysis

Data are presented as the mean ± standard deviation or as the median (first and third quartiles), where appropriate. Normality was assessed using the Shapiro-Wilk test. Continuous variables were compared using Student’s t-test for approximately normally distributed data and the Mann–Whitney U test for non-normally distributed data. Categorical variables were compared using the χ² test or Fisher’s exact test, as appropriate. Statistical analyses were performed using R software (version 4.4.0). A P-value of less than 0.05 was considered statistically significant. Receiver operating characteristic (ROC) analysis was performed to evaluate the ability of plasma HDAC1 to discriminate immune-tolerant (IT) from non-immune-tolerant (NIT) outcomes, and the area under the curve (AUC) was reported with 95% confidence intervals calculated using DeLong’s method. The optimal cutoff value was determined by maximizing the Youden index (sensitivity + specificity − 1).

## 3 Results

### 3.1 Patient selection and withdrawal outcomes

**Figure 3:**
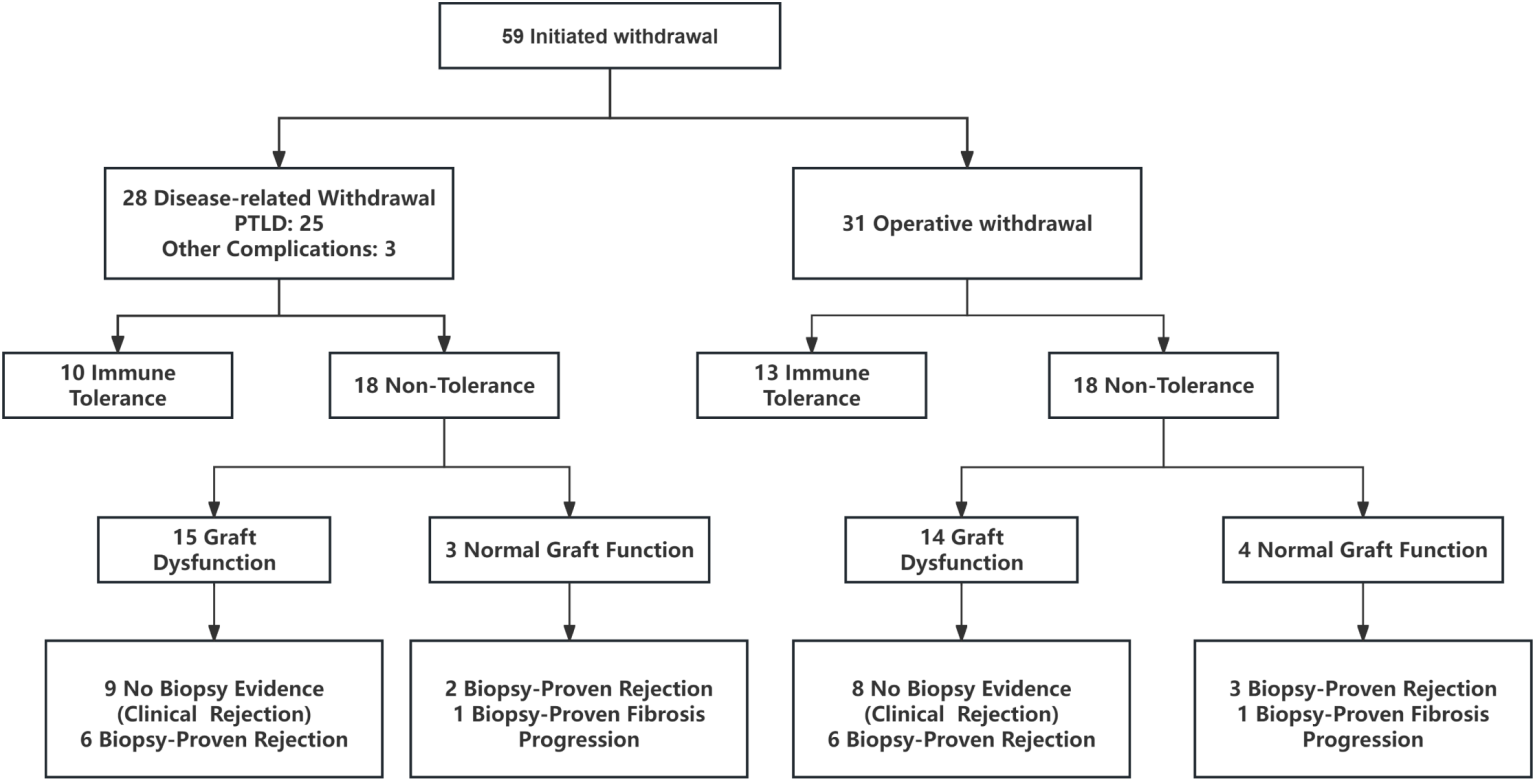
Overview of immunosuppressant withdrawal outcomes in pediatric liver transplant recipients.

Between January 1, 2017 and June 30, 2025, a total of 77 pediatric liver transplant recipients were enrolled in the immunosuppressant withdrawal cohort at Tianjin First Central Hospital. 49 patients entered planned tapering and withdrawal, and 28 underwent disease-driven dose reduction due to PTLD (n = 25) or other complications (n = 3). By the follow-up cut-off date (June 30, 2025), 59 recipients had reached the outcome adjudication time point, while 18 remained under ongoing tapering and/or follow-up (**Figure 3**; **Supplementary Figure 1**).

Among the 59 recipients who initiated withdrawal, 31 underwent planned withdrawal and 28 underwent disease-driven reduction (**Figure 3**). In the disease-driven group, 10/28 (35.7%) achieved immune tolerance, whereas 18/28 (64.3%) did not. Of the 18 non-tolerant recipients, 15 developed graft dysfunction, including 9 with clinical rejection without biopsy evidence and 6 with biopsy-proven rejection; the remaining 3 maintained normal graft function but had protocol-biopsy findings of biopsy-proven rejection (n = 2) or fibrosis progression (n = 1).

In the planned withdrawal group, 13/31 (41.9%) achieved immune tolerance and 18/31 (58.1%) did not. Among the 18 non-tolerant recipients, 14 developed graft dysfunction (8 clinical rejection without biopsy evidence; 6 biopsy-proven rejection), while 4 maintained normal graft function but showed biopsy-proven rejection (n = 3) or fibrosis progression (n = 1).

Overall, 23/59 (39.0%) achieved immune tolerance. The remaining 36/59 (61.0%) were classified as non-tolerant based on graft dysfunction or biopsy-proven rejection or fibrosis progression (**Figure 3**). All recipients who developed liver function abnormalities or adverse histology after withdrawal recovered graft function after resumption of immunosuppressive therapy.

Baseline characteristics of the planned withdrawal cohort are summarized in **Table 1**. Thirty-one pediatric liver transplant recipients were included (IT, n=13; NIT, n=18). Biliary atresia was the main indication (29/31, 93.5%). The median age at transplantation was 6.93 months (6.40, 8.27). At trial entry, recipients were a median of 67.37 months old (53.85, 76.56) and 59.90 months post-transplant (46.60, 65.67). Living-donor liver transplantation accounted for 80.7% of cases (25/31) and did not differ between IT and NIT groups (92.3% *vs* 72.2%, P = 0.359). Previous rejection was uncommon (2/31, 6.5%), and HLA-DSA were detected in 9.7% (class I) and 19.4% (class II). Baseline liver biochemistry, tacrolimus dose and trough concentration, and peripheral immune cell distributions (neutrophils, lymphocytes, CD3+, CD4+, CD8+) were comparable between groups (all P > 0.05), although tacrolimus trough concentration showed a non-significant trend toward lower levels in the IT group (median 1.00 *vs* 2.30 ng/mL, P = 0.082).

**Table 1:** Characteristics of 31 Patients Undergoing planned (protocol-driven) withdrawal.

Characteristics	Total (n = 31)	IT (n = 13)	NIT (n = 18)	P value
Donor				
Age (years)	30.08 (26.03, 36.00)	34.08(27.16, 36.00)	29.90(22.33, 36.00)	0.326
Male gender (%)	14 (45.16%)	4 (30.77%)	10 (55.56%)	0.275
Height (cm)	164.00 (159.00, 171.00)	163.00 (160.00, 170.00)	166.50 (158.50, 171.50)	0.748
Weight (kg)	59.20 (55.00, 73.50)	59.20 (50.00, 63.00)	62.00 (55.25, 75.00)	0.561
**Type (%)**				0.359
LDLT	25 (80.65%)	12 (92.31%)	13 (72.22%)	
DDLT	6 (19.35%)	1 (7.69%)	5 (27.78%)	
Recipient				
Age at transplant (months)	6.93 (6.40, 8.27)	6.77 (6.33, 7.87)	7.43 (6.55, 9.74)	0.193
Male gender (%)	14 (45.16%)	6 (46.15%)	8 (44.44%)	0.621
Height (cm)	64.00 (62.00, 67.50)	64.00 (62.50, 67.50)	65.00 (62.00, 68.50)	0.872
Weight (kg)	7.40 (6.74, 8.00)	7.20 (6.60, 7.95)	7.50 (6.79, 8.00)	0.335
Child-Pugh scores	9.00 (7.50, 10.00)	9.00 (7.00, 9.00)	9.00 (8.00, 10.00)	0.373
PELD scores	18.52 ± 8.32	17.69 ± 7.25	19.11 ± 9.18	0.635
Transplant indication (%)				0.838
Biliary atresia	29 (93.54%)	13 (100.00%)	16 (88.88%)	
Choledochal Cyst	1(3.23%)	0	1(5.56%)	
Congenital Biliary Dilatation	1(3.23%)	0	1(5.56%)	
Transplant				
Graft type (%)				0.621
Whole liver	4 (12.90%)	1 (7.69%)	3 (16.67%)	
Partial liver	27 (87.10%)	12 (92.31%)	15 (83.33%)	
Blood type combination (%)				0.262
Identical	22 (70.97%)	11 (84.62%)	11 (61.11%)	
Compatible	6 (19.35%)	2 (15.38%)	4 (22.22%)	
Incompatible	3 (9.68%)	0	3 (16.67%)	
Baseline (At trial entry)				
Age (months)	67.37 (53.85, 76.56)	67.37 (53.87, 70.20)	68.95 (54.88, 89.32)	0.567
Time since transplant (months)	59.90 (46.60, 65.67)	60.60 (48.37, 63.33)	55.6 (42.4, 70.8)	0.854
Previous Rejection Episodes (%)	2 (6.45%)	1 (7.69%)	1 (5.56%)	0.816
HLA Class I DSA (%)	3 (9.68%)	1 (7.69%)	2 (11.11%)	1.000
HLA Class II DSA (%)	6 (19.35%)	1 (7.69%)	5 (27.78%)	0.359
Extended-release tacrolimus (%)	9 (29.03%)	3 (23.08%)	6 (33.33%)	0.696
Tacrolimus trough concentration (ng/mL)	1.70 (0.90, 2.65)	1.00 (0.80, 1.70)	2.30 (1.13, 2.93)	0.082
Tacrolimus dose (mg/kg/day)	0.03 (0.02, 0.04)	0.03 (0.02, 0.03)	0.03 (0.02, 0.05)	0.170
ALT (U/L)	17.00 (13.15, 22.85)	16.40 (13.00, 20.30)	18.15 (13.38, 23.25)	0.489
AST (U/L)	30.80 (25.45, 33.50)	30.80 (26.80, 31.90)	30.95 (25.43, 33.58)	0.798
GGT (U/L)	12.00 (10.00, 15.00)	10.00 (10.00, 12.00)	12.00 (10.00, 16.75)	0.140
ALP (U/L)	261.76 ± 66.61	281.85 ± 74.20	247.26 ± 58.42	0.176
Percentage of neutrophils, %	44.00 ± 11.70	42.10 ± 8.80	45.40 ± 13.50	0.446
Percentage of lymphocytes, %	44.40 ± 10.90	46.50 ± 9.40	42.90 ± 11.90	0.374
Total T cell (CD3+), %	63.81 ± 8.74	63.13 ± 4.54	64.31 ± 10.94	0.684
CD4+ T cell, %	32.13 ± 7.34	32.70 ± 7.95	31.71 ± 7.08	0.724
CD8+ T cell, %	24.90 ± 6.35	25.27 ± 4.78	24.64 ± 7.40	0.776

### 3.2 Proteomic findings

To explore baseline molecular features associated with successful immunosuppression withdrawal, we performed plasma proteomics in 10 recipients undergoing planned tapering, including 5 who ultimately achieved immune tolerance (IT) and 5 classified as non-immune-tolerant (NIT) based on follow-up histology and/or clinical course. Within the NIT group, one patient maintained normal liver biochemistry at enrollment but showed T cell-mediated rejection on the 1-year protocol biopsy. The remaining four developed liver dysfunction during follow-up; one had biopsy-proven T cell–mediated rejection, and three recovered after returning to the pre-withdrawal tacrolimus dose and were managed as clinical rejection. Baseline plasma collected at trial entry was used for proteomic profiling.

**Figure 4:**
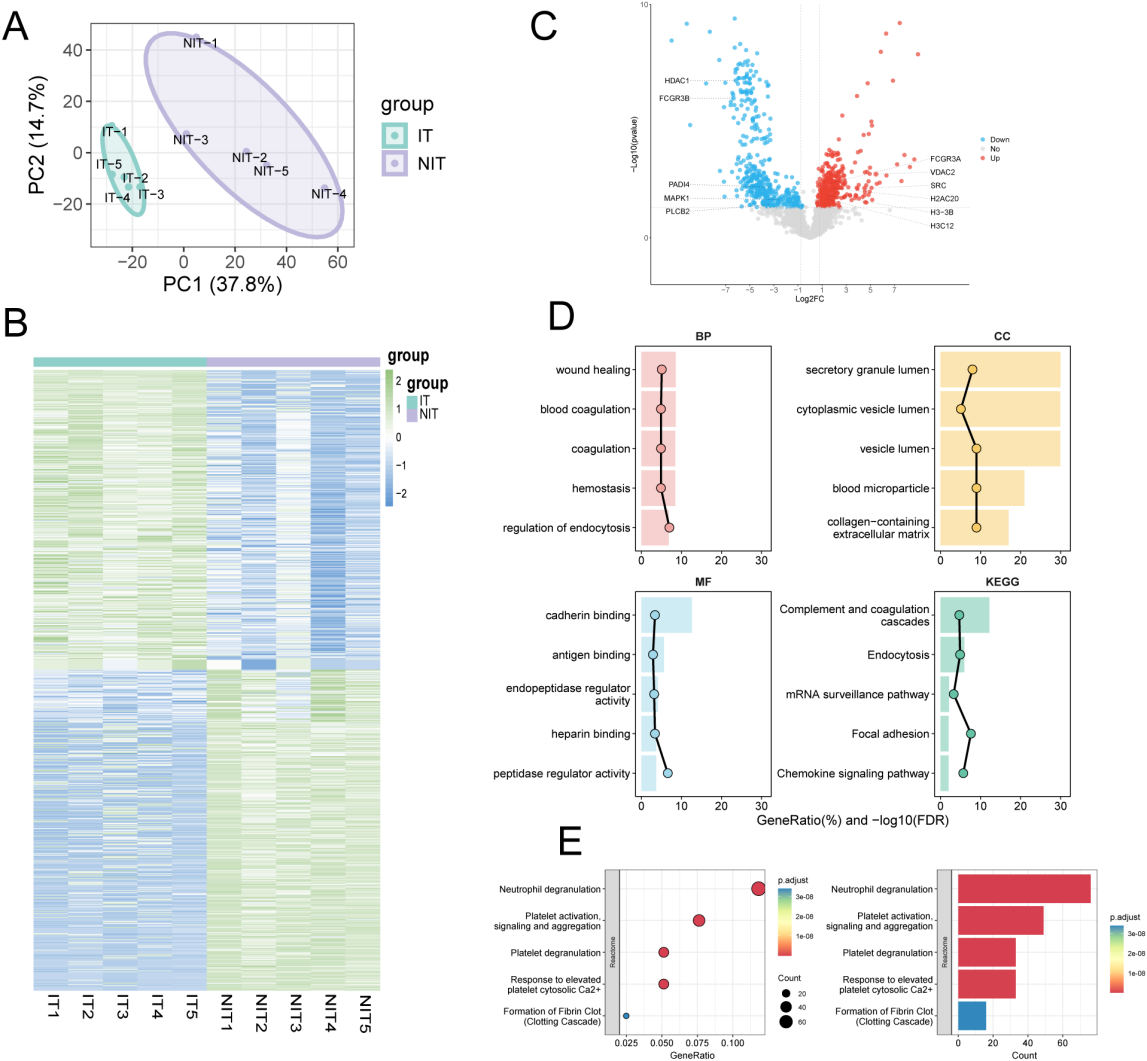
Baseline plasma proteomics differentiates immune-tolerant and non-tolerant recipients during planned immunosuppression withdrawal. **(A)** Principal component analysis (PCA) of baseline plasma proteomes from immune-tolerant (IT, n = 5) and non-immune-tolerant (NIT, n = 5) recipients. **(B)** Unsupervised hierarchical clustering heatmap showing relative abundance patterns of quantified proteins across samples. **(C)** Volcano plot of differential protein expression between IT and NIT at baseline; differential expression was defined by fold change > 1.5 and P < 0.05, yielding 802 DEPs (382 upregulated and 420 downregulated in IT *vs* NIT); HDAC1 is highlighted among downregulated proteins. **(D)** Top enriched Gene Ontology terms (BP, CC, MF) and KEGG pathways for DEPs. **(E)** Reactome pathway enrichment highlighting the top-ranked pathways, including neutrophil degranulation. IT, immune tolerance; NIT, non-immune tolerance; DEP, differentially expressed protein; GO, Gene Ontology; KEGG, Kyoto Encyclopedia of Genes and Genomes; FDR, false discovery rate.

As shown in **Figure 4**, the IT and NIT groups displayed distinct baseline proteomic patterns. Unsupervised principal component analysis showed clear group separation, indicating distinct baseline proteomic patterns between recipients who later achieved immune tolerance and those who did not (**Figure 4A**). Consistently, hierarchical clustering of the quantified proteins demonstrated a coherent group-wise segregation (**Figure 4B**), supporting systematic differences rather than isolated outliers.

Differential expression analysis identified 802 differentially expressed proteins (DEPs), including 382 proteins increased and 420 proteins decreased in IT compared with NIT (**Figure 4C**). HDAC1 was among the proteins reduced in IT. To contextualize these changes, enrichment analysis was performed using GO and KEGG annotations. The top enriched terms clustered around processes and compartments relevant to inflammatory effector programs and extracellular vesicle-associated components, alongside signaling modules linked to immune activation (**Figure 4D**). Reactome pathway analysis further prioritized neutrophil-associated biology, with neutrophil degranulation ranking among the leading pathways (**Figure 4E**). Together, these baseline proteomic features support a practical view that innate immune activity-captured here by neutrophil-related protein patterns-tracks with subsequent withdrawal outcomes, without implying causality.

**Figure 5:**
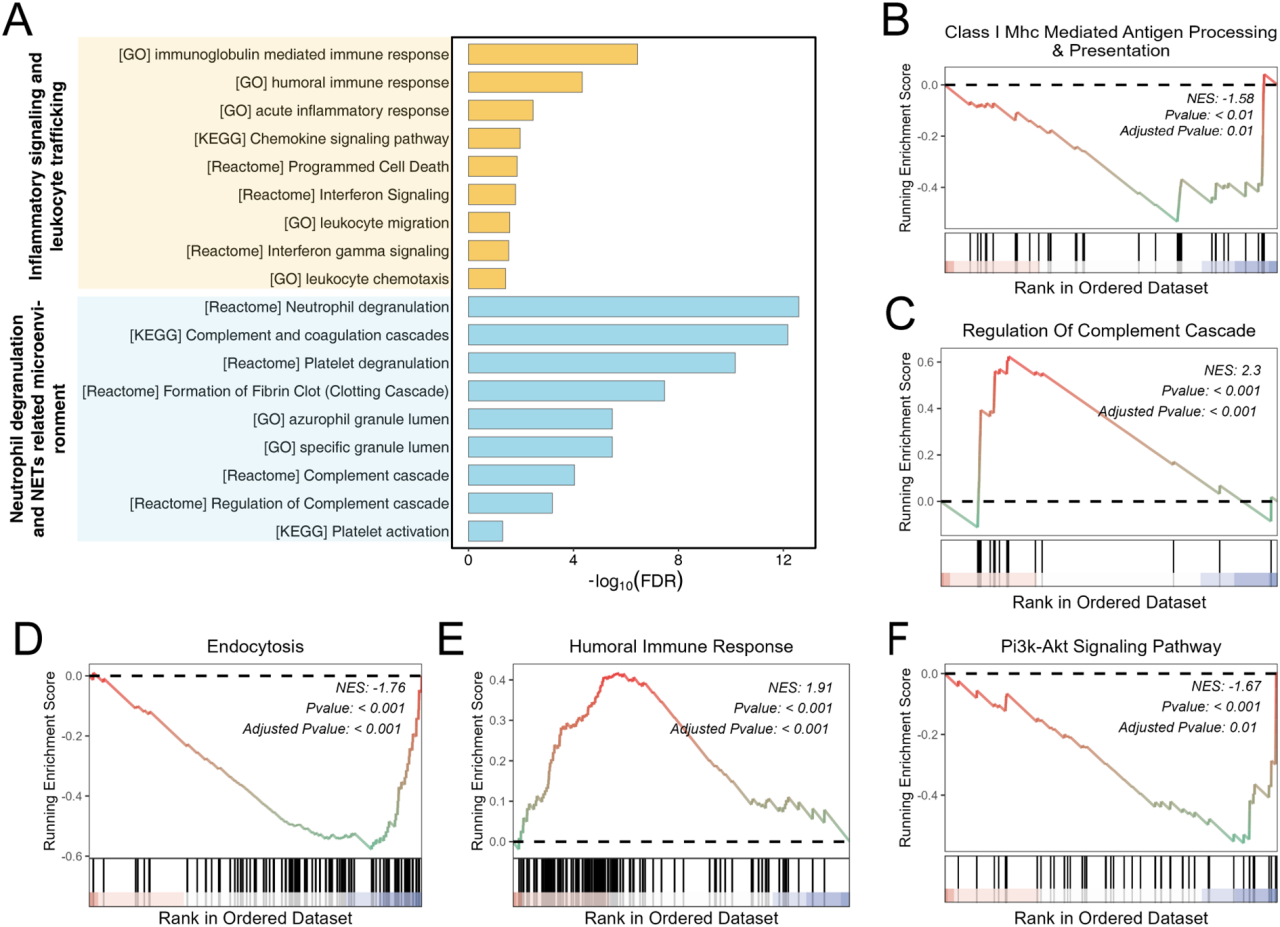
Pathway-level characterization of baseline plasma proteome differences between immune-tolerant (IT) and non-immune-tolerant (NIT) recipients. **(A)** Over-representation enrichment analysis of differentially expressed proteins (DEPs) using GO, KEGG, and Reactome databases. Bars indicate the significance of enriched terms expressed as −log10(FDR). Enriched pathways were summarized into two major modules: inflammatory signaling and leukocyte trafficking (upper) and a neutrophil degranulation/NETs-related microenvironment with complement–coagulation and platelet-associated programs (lower). **(B-F)** Gene set enrichment analysis (GSEA) based on the ranked protein list (IT vs. NIT). Representative enriched gene sets are shown, including Class I MHC-mediated antigen processing and presentation **(B)**, regulation of complement cascade **(C)**, endocytosis **(D)**, humoral immune response **(E)**, and PI3K-Akt signaling pathway **(F)**. The normalized enrichment score (NES), nominal P value, and adjusted P value are indicated in each panel. NES < 0 indicates relative down-enrichment in IT, whereas NES > 0 indicates relative up-enrichment in IT.

To interpret the biological context of the baseline plasma proteome differences between immune-tolerant (IT) and non-immune-tolerant (NIT) recipients, we performed pathway-level analyses based on the differentially expressed proteins and ranked-protein enrichment. Over-representation analysis across GO, KEGG, and Reactome highlighted two dominant themes (**Figure 5A**). The first clustered around inflammatory signaling and leukocyte trafficking, including immunoglobulin-mediated and humoral immune responses, acute inflammatory response, chemokine signaling, interferon signaling, and leukocyte migration or chemotaxis. The second theme was centered on a neutrophil-associated microenvironment, with strong enrichment for neutrophil degranulation, complement and coagulation cascades, platelet degranulation or activation, fibrin clot formation, and granule lumen terms.

We then applied gene set enrichment analysis (GSEA) using the full ranked protein list to capture coordinated pathway shifts that may not be apparent from single proteins alone (**Figures 5B–F**). Compared with NIT, IT showed reduced enrichment of pathways related to antigen processing and presentation via MHC class I (NES = −1.58), endocytosis (NES = −1.76), and PI3K-Akt signaling (NES = −1.67), whereas regulation of the complement cascade (NES = 2.30) and humoral immune response (NES = 1.91) were enriched in IT (all adjusted P ≤ 0.01 as shown). Taken together, these data describe a baseline immune landscape in which innate immune granule or complement-coagulation processes and adaptive humoral signatures coexist, while selected antigen-handling and intracellular trafficking programs are relatively attenuated in IT. This pathway-level pattern supports the use of neutrophil associated readouts as biomarkers to inform risk stratification during immunosuppressant withdrawal, rather than as definitive evidence of a single causal mechanism.

### 3.3 HDAC1 expression and candidate biomarker potential

**Figure 6:**
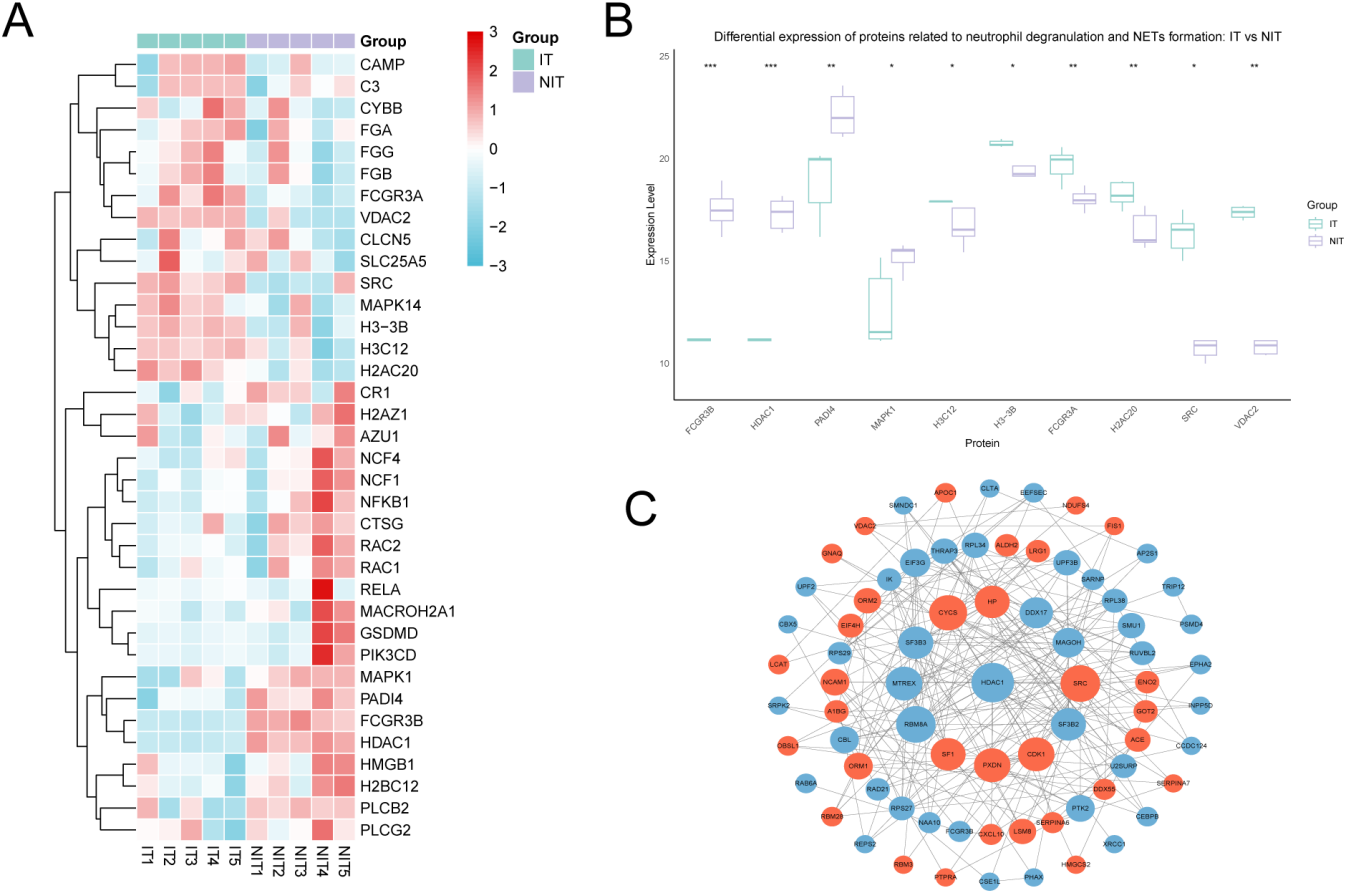
HDAC1-associated neutrophil programs differ at baseline between immune-tolerant and non-immune-tolerant recipients. **(A)** Heatmap showing baseline expression of selected proteins related to neutrophil degranulation and NET-associated pathways in the immune-tolerant (IT) and non-immune-tolerant (NIT) groups. **(B)** Bar plots of representative differentially expressed proteins highlighted from the pathway-level summary (**Figure 5A**), illustrating group-level differences between IT and NIT at baseline; HDAC1 is shown as a prioritized candidate marker. **(C)** Protein-protein interaction (PPI) network constructed from the top 100 upregulated and top 100 downregulated proteins in IT compared with NIT. Nodes represent proteins and edges represent known or predicted interactions (STRING). HDAC1 is displayed within the network to indicate its highly connected position among the differential proteome. IT, immune tolerance; NIT, non-immune tolerance; NETs, neutrophil extracellular traps; PPI, protein-protein interaction. * indicates P < 0.05, ** indicates P < 0.01, and *** indicates P < 0.001.

**Figure 6** summarizes the baseline expression patterns of proteins linked to neutrophil degranulation and NET-associated programs in immune-tolerant (IT) versus non-immune-tolerant (NIT) recipients. Several proteins within these pathways differed consistently between groups (**Figure 6A**). Among the candidates, HDAC1 showed lower abundance in the IT group, accompanied by group-level differences in FCGR3B, PADI4, and MAPK1, aligning with the neutrophil-centered signal observed at the pathway level.

To make the group contrast more explicit, the proteins highlighted in the pathway summary (**Figure 5A**) were displayed as bar plots (**Figure 6B**). HDAC1 demonstrated one of the clearest separations between IT and NIT at baseline, supporting its prioritization as a candidate biomarker for stratifying patients during immunosuppressant tapering rather than as definitive evidence of causality.

We further evaluated network context using a protein-protein interaction (PPI) analysis constructed from the top 100 upregulated and downregulated proteins (**Figure 6C**). Within this network, HDAC1 occupied a highly connected position with multiple interactions, indicating that it sits in a dense regulatory neighborhood of the differential proteome. This hub-like placement strengthens the rationale to track HDAC1 as an integrated readout of baseline immune state when planning and monitoring immunosuppressant withdrawal.

**Table 2** summarizes baseline donor, recipient, and transplant characteristics in the ELISA validation cohort (n = 39), comprising immune-tolerant (IT, n = 17) and non-immune-tolerant (NIT, n = 22) recipients undergoing immunosuppression withdrawal. Donor age was comparable between groups, and approximately half of donors were male (53.6%). Most recipients underwent transplantation in infancy, and biliary atresia remained the leading indication (35/39, 89.7%). Living donor liver transplantation predominated (34/39, 87.2%), and graft type and ABO compatibility distributions did not differ materially between IT and NIT.

**Table 2:** Characteristics of 39 patients in the elisa validation cohort undergoing immunosuppression withdrawal.

Characteristics	Total (n = 39)	IT (n = 17)	NIT (n = 22)	P value
Donor				
Age (years)	30.08 (26.53, 36.45)	32.49 (27.16, 36.00)	29.95 (25.29, 37.30)	0.650
Male gender(%)	21 (53.85%)	7(41.18%)	14 (63.64%)	0.284
Height (cm)	168.00 (161.75, 170.00)	163.00 (160.00, 170.00)	169.00 (163.00, 171.50)	0.400
Weight (kg)	62.00 (55.00, 71.50)	61.50 (50.00, 70.00)	66.00 (55.25, 75.00)	0.364
Type (%)				1.000
LDLT	34 (87.18%)	15 (88.24%)	19 (86.36%)	
DDLT	5 (12.82%)	2 (11.76%)	3 (13.64%)	
Recipient				
Age at transplant (months)	7.10 (6.40, 9.16)	6.77 (6.33, 7.87)	7.43 (6.55, 10.50)	0.229
Male gender(%)	19 (48.72%)	7 (41.18%)	12 (54.55%)	0.613
Height (cm)	64.00 (62.30, 68.00)	64.00 (63.00, 68.00)	64.00 (62.00, 67.75)	0.921
Weight (kg)	7.40 (6.60, 8.00)	7.20 (6.50, 8.00)	7.45 (6.72, 8.00)	0.532
Child-Pugh scores	9.00 (7.00, 10.00)	8.00 (7.00, 9.00)	9.00 (7.00, 10.00)	0.475
PELD scores	18.59 ± 8.07	17.18 ± 6.53	19.68 ± 9.09	0.323
Transplant indication (%)				0.495
Biliary atresia	35 (89.70%)	17 (100.00%)	18 (81.80%)	
Cholestasis	2 (5.10%)	0	2 (9.10%)	
Choledochal Cyst	1 (2.60%)	0	1 (4.50%)	
Biliary Complications After Liver Transplantation	1 (2.60%)	0	1 (4.50%)	
Transplant				
Graft type (%)				1.000
Whole liver	3 (7.69%)	1 (5.88%)	2 (9.09%)	
Partial liver	36 (92.31%)	16 (94.12%)	20 (90.91%)	
Blood type combination (%)				0.352
Identical	31 (79.50%)	15 (88.20%)	16 (72.70%)	
Compatible	5 (12.80%)	2 (11.80%)	3 (13.60%)	
Incompatible	3(7.70%)	0	3 (13.60%)	
Baseline (At trial entry)				
Reasons for IW				0.548
Planned (%)	27 (69.23%)	13 (76.47%)	14 (63.64%)	
PTLD (%)	10 (25.64%)	4 (23.53%)	6 (27.27%)	
Adverse Effects (%)	2 (5.13%)	0	2 (9.09%)	
Age (months)	61.76 (48.52, 71.57)	61.90 (48.17, 69.90)	61.43 (49.83, 75.93)	0.723
Time since transplant (months)	54.33 (42.17, 63.68)	55.03 (42.27, 62.33)	52.81 (42.88, 64.28)	0.955
Previous Rejection Episodes (%)	3 (7.69%)	1 (5.88%)	2 (9.09%)	1.000
HLA Class I DSA (%)	2 (5.13%)	1 (5.88%)	1 (4.55%)	1.000
HLA Class II DSA (%)	8 (20.51%)	2 (11.76%)	6 (27.27%)	0.426
Extended-release tacrolimus (%)	9 (23.08%)	3 (17.65%)	6 (27.27%)	0.704
Tacrolimus trough concentration (ng/mL)	1.20 (0.80, 2.55)	1.00 (0.70, 1.70)	1.50 (1.00, 2.68)	0.178
Tacrolimus dose (mg/kg/day)	0.03 (0.02, 0.04)	0.03 (0.02, 0.03)	0.03 (0.02, 0.04)	0.079
ALT (U/L)	17.00 (12.85, 23.80)	16.40 (13.00, 20.30)	19.40 (12.25, 24.60)	0.424
AST (U/L)	31.00 (26.15, 37.05)	30.80 (26.15, 40.65)	31.95 (26.80, 39.50)	0.566
GGT (U/L)	12.00 (10.00, 14.50)	10.00 (9.00, 13.00)	12.30 (10.00, 14.75)	0.185
ALP (U/L)	262.15 ± 79.01	276.65 ± 72.82	250.94 ± 83.38	0.312
Percentage of neutrophils, %	42.78 ± 10.42	43.69 ± 8.69	42.08 ± 11.73	0.624
Percentage of lymphocytes, %	45.28 ± 10.74	44.34 ± 10.01	46.01 ± 11.45	0.631
Total T cell (CD3+), %	65.81 ± 10.35	66.69 ± 9.50	65.14 ± 11.13	0.643
CD4+ T cell, %	31.49 ± 7.16	33.33 ± 7.20	30.06 ± 6.96	0.162
CD8+ T cell, %	28.06 ± 9.05	27.79 ± 7.35	28.27 ± 10.33	0.867
HDAC1 in Plasma (pg/ml)	1550.00 (718.70, 4327.96)	784.10 (552.10, 1453.20)	3987.63 (1390.26, 6448.67)	0.002

At trial entry, the clinical context for withdrawal was similar across groups, with planned withdrawal accounting for 69.2% of cases, followed by PTLD-related reduction (25.6%) and withdrawal driven by adverse effects (5.1%). The median time from transplantation to trial entry was 54.3 months, without group differences. Baseline liver biochemistry (ALT, AST, GGT, and ALP), weight-adjusted tacrolimus dose, and routine immune profiling (neutrophil and lymphocyte percentages; CD3+, CD4+, and CD8+ T cell proportions) were broadly comparable between IT and NIT (all P > 0.05). Baseline tacrolimus trough concentration was also comparable between groups (IT: 1.00 ng/mL (0.70, 1.70) *vs* NIT: 1.50 ng/mL (1.00, 2.68); P = 0.178). In contrast, baseline plasma HDAC1 levels differed substantially: IT recipients showed lower concentrations than NIT recipients (784.10 pg/ml (552.10, 1453.20) *vs* 3987.63 pg/ml (1390.26, 6448.67), P = 0.002). This separation at baseline supports HDAC1 as a candidate marker for stratifying the likelihood of successful withdrawal, rather than reflecting differences in conventional liver tests or routine lymphocyte subsets at trial entry. To account for potential confounding by tacrolimus exposure, we performed multivariable logistic regression including baseline plasma HDAC1 and tacrolimus trough concentration. Baseline HDAC1 remained independently associated with withdrawal failure after adjustment (adjusted OR = 1.001, 95% CI 1.000 – 1.002; P = 0.025; **Supplementary Table 2**).

**Figure 7:**
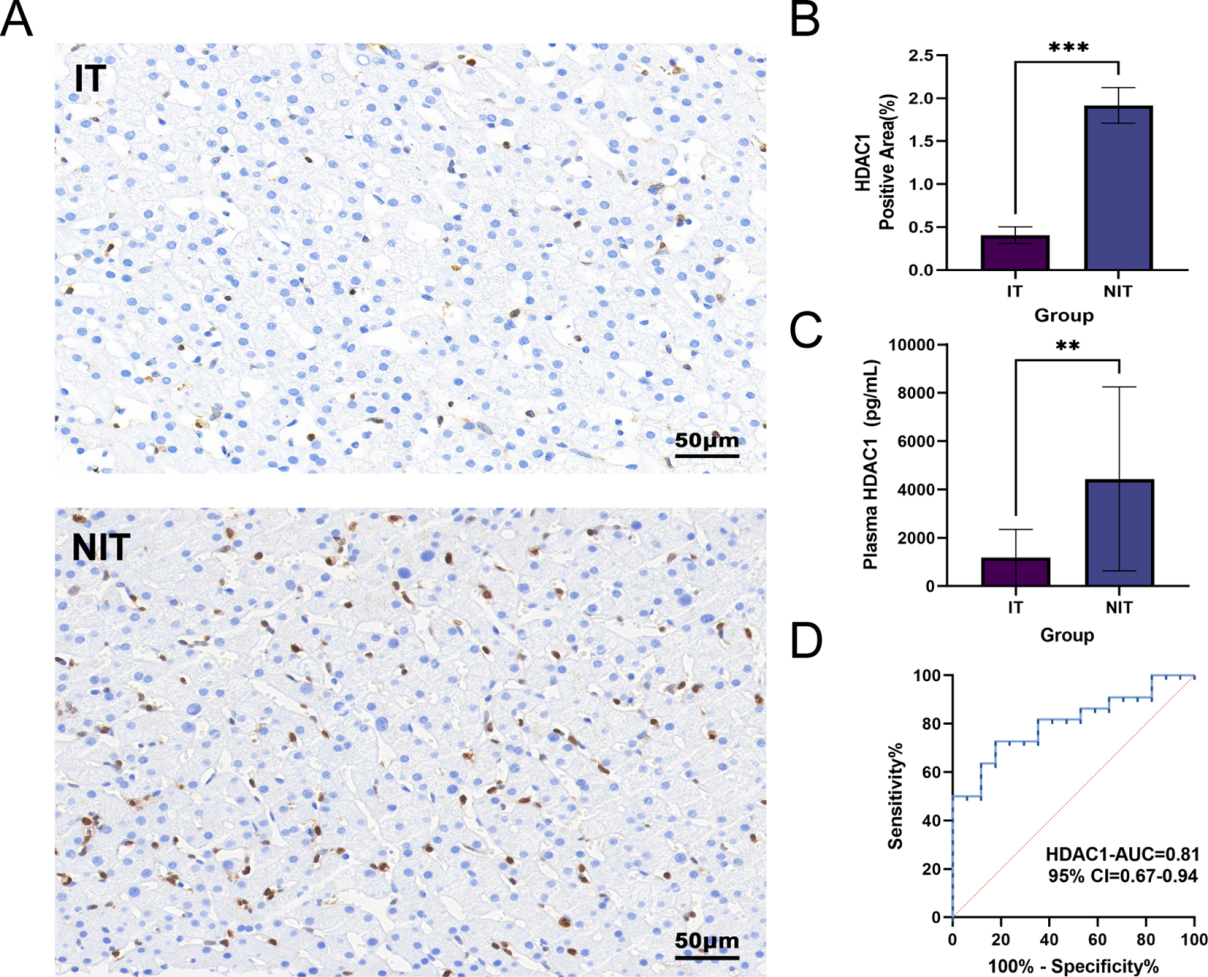
Baseline hepatic and plasma HDAC1 levels and discriminative performance for immune tolerance during immunosuppression withdrawal. **(A)** Representative immunohistochemistry (IHC) staining of HDAC1 in baseline protocol liver biopsy sections from immune-tolerant (IT, n = 5) and non-immune-tolerant (NIT, n = 5) pediatric liver transplant recipients. Scale bar, 50 μm. **(B)** Quantification of hepatic HDAC1 staining expressed as HDAC1-positive area fraction (Area%). Each dot represents one patient; horizontal bars indicate median with interquartile range. IT showed lower hepatic HDAC1 staining than NIT (P = 0.0004). **(C)** Plasma HDAC1 concentrations at trial entry measured by ELISA in the validation cohort (IT, n = 17; NIT, n = 22). Data are shown as median with interquartile range; each dot represents one patient. Plasma HDAC1 was lower in IT than NIT (P = 0.002). **(D)** ROC curve of plasma HDAC1 for discriminating immune tolerance; AUC = 0.81 (95% CI 0.67-0.94), P = 0.002. The optimal cutoff was 1565 pg/mL (Youden index), with sensitivity 72.73% and specificity 82.35%. Statistics: Two-group comparisons in **(B)** and **(C)** were performed using a two-tailed unpaired Mann-Whitney U test. ROC significance is reported in **(D)**.

To corroborate the clinical relevance of HDAC1 at baseline, we assessed HDAC1 expression in baseline liver biopsy specimens and paired plasma collected at trial entry. Representative IHC images (IT, n = 5; NIT, n = 5) showed weaker HDAC1 staining in IT compared with NIT (**Figure 7A**), supported by quantitative image analysis demonstrating a lower HDAC1-positive area fraction in IT (**Figure 7B**). Consistently, plasma HDAC1 concentrations at trial entry were lower in IT than in NIT (**Figure 7C**). In ROC analysis, baseline HDAC1 discriminated IT from NIT with an AUC of 0.81 (**Figure 7D**). Using the Youden index, the balanced cutoff for plasma HDAC1 was 1565 pg/mL, yielding a sensitivity of 72.7% and specificity of 82.4%. Sensitivity analysis excluding ABO-incompatible recipients (n = 3) showed similar discrimination (AUC = 0.82; **Supplementary Figure 2**). Taken together, tissue and plasma based assessments converge on the same direction of association and support baseline HDAC1 as a practical marker for risk stratification.

## 4 Discussion

Immunosuppression withdrawal after pediatric liver transplantation remains a high-value but high-risk clinical strategy. The practical problem is not whether tolerance exists, but how to identify candidates who can proceed safely, limit avoidable rejection, and reduce infectious and drug-related complications during tapering (4, 16). Large pediatric withdrawal studies have shown that carefully selected recipients can discontinue calcineurin inhibitors, yet outcomes are heterogeneous and prediction remains imprecise. Biomarkers that support withdrawal planning and monitoring are still needed in routine practice (6, 7, 17).

This study links baseline circulating and intrahepatic HDAC1 with withdrawal outcomes and places neutrophil-related programs upstream in the biomarker landscape. In our planned withdrawal cohort, baseline clinical indices and conventional immune cell proportions did not clearly distinguish tolerant from non-tolerant patients at trial entry. By contrast, proteomic profiling at baseline separated IT and NIT recipients and repeatedly pointed to neutrophil biology. In the independent ELISA cohort, plasma HDAC1 was lower in IT than NIT, and this directionality was mirrored in baseline liver biopsy specimens by immunohistochemistry. The convergence across plasma proteomics, plasma ELISA, and tissue staining supports HDAC1 as a reproducible marker in our setting rather than an isolated discovery signal.

The pathway patterns provide a clinically interpretable frame without forcing a mechanism. Differential-protein pathway mapping highlighted two dominant themes: inflammatory signaling/leukocyte trafficking and a neutrophil degranulation/NETs-related microenvironment. The subsequent GSEA panels were directionally consistent, with negative NES values indicating relative downregulation in IT compared with NIT. Taken together, the tolerant phenotype at baseline appears less dominated by inflammatory recruitment and neutrophil activation programs, even before dose reduction begins. This aligns with the concept that innate immune tone sets the “starting condition” for tapering and may shape how the graft and host respond when immunosuppression pressure is reduced (18, 19). Similar emphasis on innate immune cell trafficking in the liver has been described in transplant immunology more broadly (12, 13, 20).

NET biology in transplantation is commonly discussed in the context of injury and rejection, but the literature is not one-directional. NET fragments can amplify innate immune activation and impair tolerance in experimental models (21–23), and clinical liver transplantation studies have connected circulating cell-free DNA/NET-related signals with perioperative inflammation and outcomes (15, 24–26). Mechanistic studies in liver transplantation suggest that NETosis susceptibility is shaped by defined neutrophil regulatory circuits (27), and that NET-derived signals can further amplify innate immune activation while reinforcing antigen-presenting pathways that may undermine tolerance (28). Our data do not prove that NETs drive tolerance failure, and the proteomic approach cannot localize the source of each protein signal. Specifically, while our findings highlight the association between HDAC1 and neutrophil-associated signatures, we cannot exclude the contribution of other cell types, such as hepatocytes or other infiltrating immune cells, to the circulating HDAC1 pool, as HDAC1 is a ubiquitously expressed nuclear enzyme released during cellular turnover or stress. What it does suggest is simpler: recipients who ultimately tolerate withdrawal present with a baseline proteomic state that is less enriched for neutrophil effector programs, including pathways labeled as neutrophil degranulation and NET-associated signatures. That observation is compatible with a lower inflammatory setpoint rather than a single pathway switch (14, 21, 29).

HDAC1 emerged as the most clinically actionable marker in our dataset because it can be measured in plasma and is detectable in baseline liver tissue. HDAC1 participates in epigenetic regulation and has been linked to inflammatory signaling in non-transplant contexts, but translating those roles directly to pediatric liver allografts would be speculative (30). In our cohort, the association was consistent and clinically oriented: lower baseline HDAC1 marked recipients more likely to achieve immune tolerance during withdrawal. ROC analysis supported discrimination, and the Youden-index cutoff (1565 pg/mL) provided a pragmatic threshold with balanced sensitivity and specificity. This type of threshold is not a substitute for clinical judgment, protocol biopsy, or longitudinal liver tests; it is a potential tool for risk stratification at entry. In practice, a baseline marker that enriches for likely tolerant candidates could reduce exposure to unnecessary tapering attempts and limit the downstream need to rescue with intensified immunosuppression-an outcome that matters because infection risk and cumulative toxicity remain central concerns in children (4–6, 23, 31, 32). Biologically, HDAC1 also sits at a plausible junction between chromatin regulation and neutrophil effector programs, as experimental data indicate that Class I histone deacetylases, including HDAC1, can promote NET formation and downstream tissue injury, which provides a rationale for why higher baseline HDAC1 might track with a more injury-prone innate state (33). Notably, in the PPI network analysis, HDAC1 appeared as a highly connected node within the differentially represented protein network, supporting its centrality within the baseline inflammatory landscape while not establishing a mechanistic role. Taken together, our findings support baseline HDAC1 as a candidate marker to enrich patient selection and to complement monitoring strategies that track graft injury over time, aligning with current efforts to balance rejection control with preservation of protective immune function in immunosuppression management (16, 22, 34).

The work also complements, rather than displaces, the established T cell–centric tolerance literature. Transcriptional tests, donor-specific antibody profiles, and Treg-associated signals have provided important frameworks for operational tolerance in liver transplantation (9, 10, 35–37). However, these tools are not uniformly available, may require complex assays, and sometimes reflect downstream adaptive states after immunosuppression has already been modified. Our findings argue that an innate immune signature-captured here through neutrophil-associated pathway enrichment and a plasma marker-can be informative at baseline, when withdrawal decisions are first made. This is consistent with a broader shift in transplant immunology toward multi-cell interactions and early innate cues that shape adaptive trajectories (7, 8, 12, 13, 38).

Several limitations define how far the conclusions can be taken. First, this is a single-center cohort with a modest sample size, and the proteomic discovery set was deliberately small. Second, plasma HDAC1 is not cell-specific; the study does not establish which cells contribute most to the circulating signal, nor does it prove causality. Third, the withdrawal population includes both planned withdrawal and disease-related reduction in the larger cohort, which reflects real-world practice but can introduce heterogeneity in immune status and monitoring intensity. Finally, although protocol biopsies anchor outcome definitions, longer follow-up will be needed to confirm durability and to understand how baseline HDAC1 behaves across time, infections, and intercurrent inflammatory events.

The immediate next step is prospective validation integrated into the withdrawal workflow. A practical model would combine baseline HDAC1 with standard clinical eligibility, histology, and a small set of laboratory markers, then test whether the combined approach improves safety signals during tapering, including early biochemical flares and biopsy-proven rejection. Mechanistic work can proceed in parallel, but it should follow the clinical observation rather than lead it. For the “immunotherapy era” framing, the most relevant message is managerial: immune monitoring during immunosuppression minimization needs to be grounded in pathways that capture inflammatory readiness and leukocyte activation, and neutrophil-related programs appear to be part of that readiness in pediatric liver transplantation.

## 5 Conclusion

Overall, our data suggest a baseline, neutrophil-linked inflammatory signature associated with withdrawal outcomes and identify plasma HDAC1 as a measurable marker that may help risk-stratify candidates for immunosuppression minimization, potentially informing withdrawal management; however, prospective multicenter validation is required before clinical implementation.

## Data Availability

The datasets presented in this study can be found in online repositories. The names of the repository/repositories and accession number(s) can be found in the article/**Supplementary Material**.
