# Supplementary material for: Neutrophil-associated plasma proteomics identifies HDAC1 as a baseline biomarker of immune tolerance during immunosuppressant withdrawal after pediatric liver transplantation: a single-center cohort study
Source: Front Immunol. 2026 Mar 26;17:1800926. doi: 10.3389/fimmu.2026.1800926 (PMC13061671; doi:10.3389/fimmu.2026.1800926)
Supplement: Supplementary file 1 [file DataSheet1.docx]

Supplementary Material

# Supplementary Figures and Tables

1. **Supplementary Figure S1**

**Figure S1. Study flow diagram and biomarker analysis subsets**

A total of 77 pediatric liver transplant recipients entered the immunosuppression tapering/withdrawal program. By the follow-up cut-off date (June 30, 2025), 18 recipients were still undergoing stepwise tapering or were in post-withdrawal follow-up without graft dysfunction and had not yet reached the predefined time point for outcome adjudication; therefore, 59 recipients were evaluable for outcome classification. Within the evaluable cohort, 31 recipients underwent planned (protocol-driven) immunosuppression tapering and withdrawal, whereas 28 underwent disease-/event-driven immunosuppression reduction (PTLD or other clinical indications). Biomarker analyses were performed using baseline samples collected at trial entry (before tapering): discovery proteomics was conducted in 10 recipients selected from the planned cohort, ELISA validation was performed in 39 recipients with baseline plasma available, and HDAC1 immunohistochemistry was performed in baseline liver biopsy FFPE specimens from 10 recipients (5 immune-tolerant and 5 non-immune-tolerant) selected from the planned cohort according to predefined criteria.


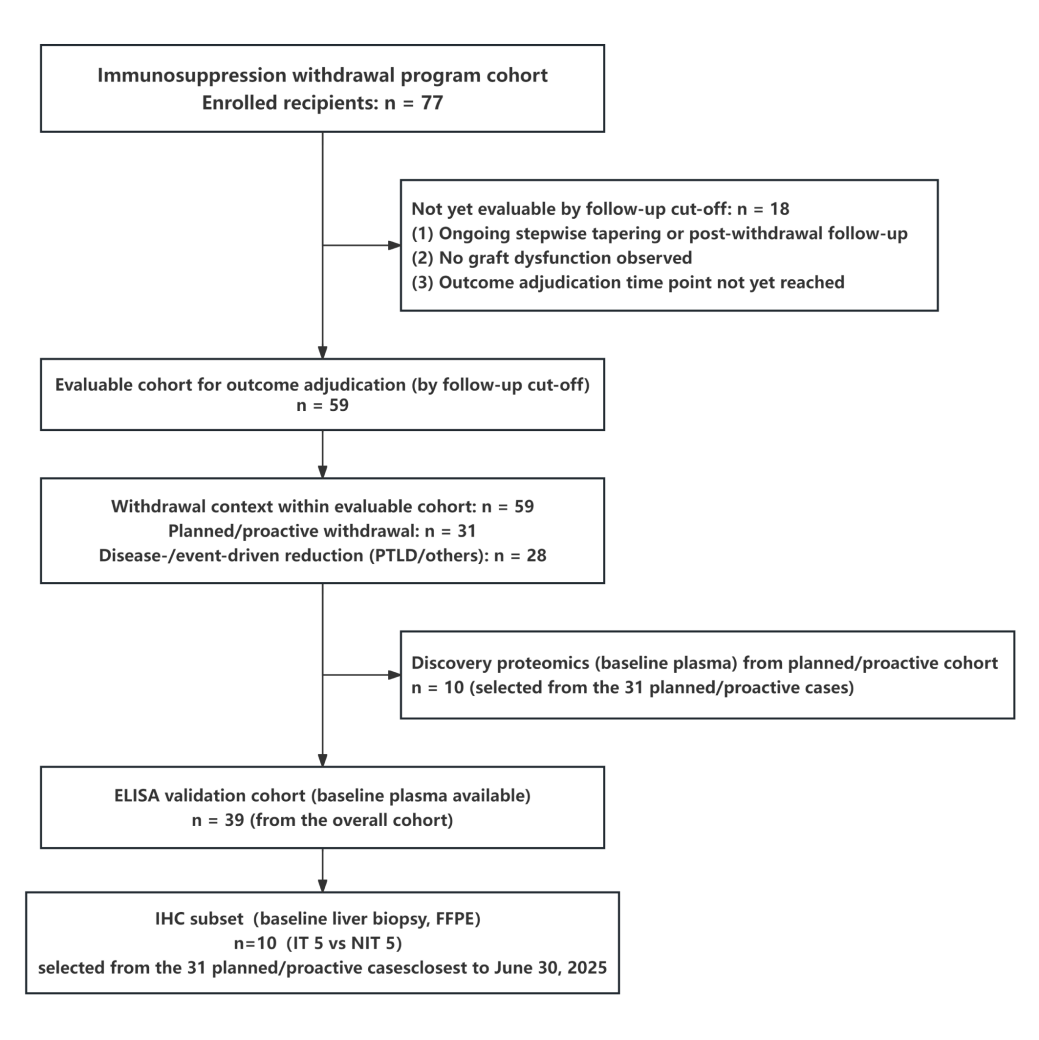


1. **Supplementary Figure S2**

**Figure S2. Sensitivity analysis excluding ABO-incompatible recipients**

1. After excluding ABO-incompatible recipients (n = 3), baseline plasma HDAC1 concentrations remained significantly lower in immune-tolerant (IT; n = 17) than in non-immune-tolerant (NIT; n = 19) recipients (784.10 (552.10, 1453.20) vs 3677.52 (1390.26, 5821.83) pg/mL; Mann-Whitney U test, P = 0.001).
2. ROC curve evaluating baseline plasma HDAC1 for discriminating IT from NIT after excluding ABO-incompatible recipients (AUC = 0.82, 95% CI 0.68–0.96; DeLong; P = 0.003).


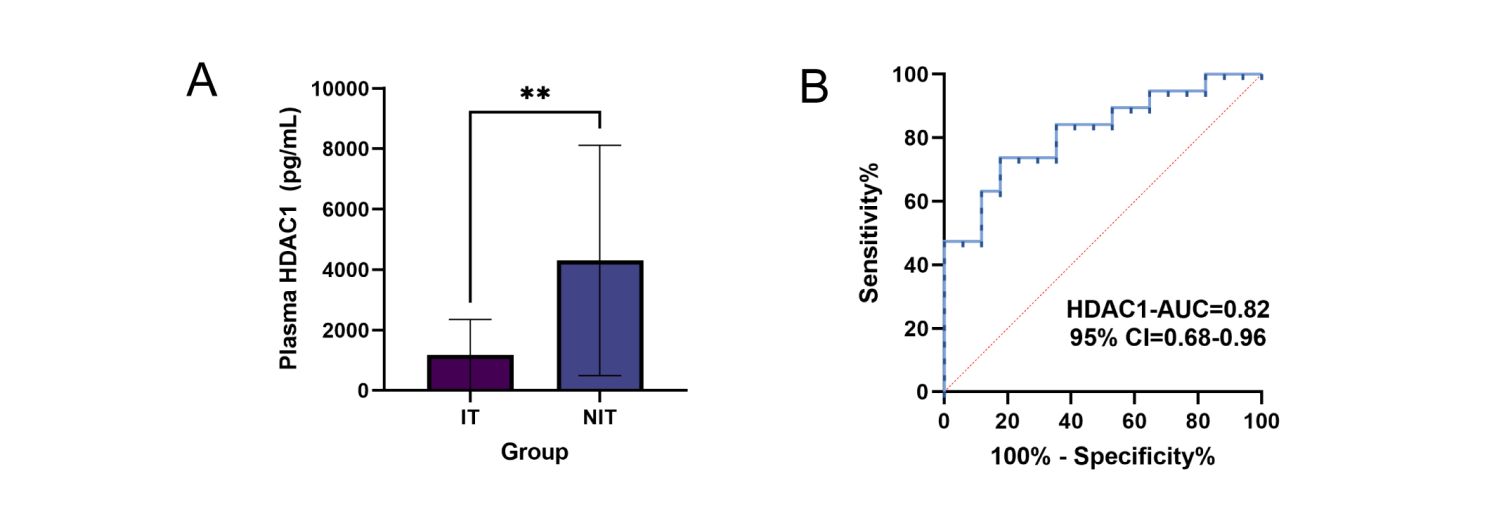


1. **Supplementary Table S1**

**Table S1 Baseline characteristics of the discovery proteomics cohort (planned withdrawal recipients; n = 10)**

| **Characteristics** | **Total (n = 10)** | **IT (n = 5)** | **NIT (n = 5)** | **P value** |
| --- | --- | --- | --- | --- |
| Donor |  |  |  |  |
| Age (years) | 32.79 (30.17, 34.33) | 34.08 (32.49, 34.41) | 30.995 (29.90，33.08) | 0.548 |
| Male gender (%) | 3 (30.00%) | 1 (20.00%) | 2 (40.00%) | 1.000 |
| Height (cm) | 163.00 (158.00, 169.00) | 163.00 (160.00, 163.00) | 165.00 (158.00, 170.00) | 0.841 |
| Weight (kg) | 58.00 (55.00, 63.00) | 56.00 (55.00, 62.00) | 59.00 (55.00, 75.00) | 0.841 |
| Type |  |  |  | 1.000 |
| LDLT (%) | 1 (10.00%) | 5 (100.00%) | 4 (80.00%) |  |
| DDLT (%) | 9 (90.00%) | 0 | 1 (20.00%) |  |
| Recipient |  |  |  |  |
| Age at transplant (months) | 6.93 (6.31, 7.76) | 6.33 (5.90, 6.77) | 7.43 (7.10, 7.90) | 0.151 |
| Male gender (%) | 6 (60.00%) | 4 (80.00%) | 2 (40.00%) | 0.524 |
| Height (cm) | 64.00 (63.00, 68.00) | 64.00 (63.00, 68.00) | 64.00 (63.00, 66.00) | 1.000 |
| Weight (kg) | 7.50 (6.80, 7.90) | 7.40 (6.80, 7.70) | 7.50 (6.80, 8.00) | 0.841 |
| Child-Pugh scores | 9.00 (8.00, 9.00) | 9.00 (9.00, 9.00) | 9.00 (7.00, 9.00) | 0.690 |
| PELD scores | 18.00 (12.00, 24.00) | 18.00 (10.00, 19.00) | 20.00 (14.00, 25.00) | 0.548 |
| Transplant indication (%) |  |  |  | 0.801 |
| Biliary atresia | 10 (100.00%) | 5 (100.00%) | 5 (100.00%) | - |
| Transplant |  |  |  |  |
| Graft type (%) |  |  |  | 1.000 |
| Whole liver | 1 (10.00%) | 0 | 1 (20.00%) |  |
| Partial liver | 9 (90.00%) | 5 (100%) | 4(80.00%) |  |
| Blood type combination (%) |  |  |  | 1.000 |
| Identical | 9 (90.00%) | 5 (100.00%) | 4 (80.00%) |  |
| Compatible | 1 (10.00%) | 0 | 1 (20.00%) |  |
| Baseline (At trial entry) |  |  |  |  |
| Age (months) | 50.70 (46.56,63.46) | 53.83 (48.17,66.67) | 48.60 (46.03,52.80) | 0.690 |
| Time since transplant (months) | 44.00 (40.55,56.80) | 47.50 (42.27,59.90) | 42.30 (38.60,45.70) | 0.548 |
| Previous Rejection Episodes (%) | 1 (10.00%) | 0 | 1 (20.00%) | 1.000 |
| HLA Class I DSA (%) | 0 | 0 | 0 | - |
| HLA Class II DSA (%) | 2 (20.00%) | 1 (20.00%) | 1 (20.00%) | 1.000 |
| Extended-release tacrolimus (%) | 1 (10.00%) | 0 | 1 (20.00%) | 1.000 |
| Tacrolimus trough concentration (ng/mL) | 1.70 (0.90, 3.15) | 2.20 (0.90, 3.50) | 1.20 (0.90, 2.70) | 0.841 |
| Tacrolimus dose ( mg/kg/day) | 0.03 (0.03, 0.03) | 0.03 (0.02, 0.03) | 0.03 (0.03, 0.04) | 0.310 |
| ALT (U/L) | 19.60 (16.80, 23.10) | 16.40 (13.90, 22.30) | 20.80 (18.30, 23.30) | 0.421 |
| AST (U/L) | 31.90 (30.90, 33.20) | 31.90 (30.80, 32.00) | 32.50 (31.40, 33.50) | 0.690 |
| GGT (U/L) | 10.00 (10.00, 12.00) | 10.00 (10.00, 11.00) | 12.00 (10.00, 14.00) | 0.421 |
| ALP (U/L) | 210.00 (195.00, 315.00) | 205.00 (177.00, 342.00) | 215.00 (198.00, 271.00) | 1.000 |
| Percentage of neutrophils, % | 44.70 (37.17, 52.58) | 46.30 (43.10, 56.80) | 38.30 (33.20, 51.30) | 0.310 |
| Percentage of lymphocytes, % | 47.15 (36.82, 54.58) | 46.30 (33.00, 48.80) | 48.00 (38.40, 57.00) | 0.421 |
| Total T cell (CD3+), % | 64.94 (62.72, 68.94) | 63.18 (62.57, 66.05) | 69.29 (63.83, 69.79) | 0.310 |
| CD4+ T cell, % | 33.55 (30.48, 38.56) | 34.53 (29.93, 41.01) | 32.58 (32.12, 38.23) | 0.841 |
| CD8+ T cell, % | 25.91 (24.45, 28.30) | 26.83 (24.31, 32.35) | 24.98 (24.86, 26.96) | 0.690 |

1. **Supplementary Table S2**

**Multivariable logistic regression for withdrawal failure adjusting for tacrolimus trough concentration in the ELISA validation cohort (n = 39)**

| **Predictor** | **β (B)** | | | **SE** | | **Wald χ²** | | **df** | | **P value** | | **Adjusted OR(Exp(B))** | | **95% CI for OR** |  |
| --- | --- | --- | --- | --- | --- | --- | --- | --- | --- | --- | --- | --- | --- | --- | --- |
| HDAC1 | 0.001 | | | 0.000 | | 5.031 | | 1 | | 0.025 | | 1.001 | | 1.000-1.002 |  |
| Tacrolimus trough concentration | 0.357 | | | 0.365 | | 0.961 | | 1 | | 0.327 | | 1.430 | | 0.700-2.922 |  |
| Intercept | | -2.268 | 0.956 | | 5.628 | | 1 | | 0.018 | | 0.104 | | - | | |

# Supplementary Methods

1. Supplementary Methods S1. Proteomics data processing, statistics, and pathway analyses

Raw DIA mass spectrometry data were processed using Spectronaut Pulsar (v18.4, Biognosys) under default settings for identification and library generation (Trypsin/P; up to two missed cleavages; carbamidomethylation as a fixed modification; oxidation (M) as a variable modification). Precursor and protein q-value cutoffs were set to 0.01 (1% FDR), and protein quantification was performed at the MS2 level with local normalization enabled in Spectronaut to reduce run-to-run technical variation.

For differential abundance testing between IT and NIT at baseline, protein quantities were log-transformed and compared using the two-sided group comparison implemented in Spectronaut to obtain nominal P values. Proteins were filtered for quantification completeness prior to testing, and missing values were not imputed. Differentially expressed proteins (DEPs) were defined as fold change > 1.5 with nominal P < 0.05. To address multiple testing across quantified proteins, Benjamini–Hochberg false discovery rate (BH-FDR)–adjusted P values were additionally calculated and reported alongside nominal P values.

Over-representation enrichment analyses (GO/KEGG/Reactome) were performed using the DEP list, with the background set defined as all quantified proteins passing filtering in the discovery dataset. Enrichment P values were corrected using BH-FDR, and pathways with FDR-adjusted P < 0.05 were considered significant. GSEA was performed using a preranked list of all quantified proteins ranked by log2 fold change (IT vs NIT) to identify coordinated pathway shifts; results were interpreted based on FDR (q values) after BH correction. Protein–protein interaction (PPI) analysis was performed using the STRING database.

The complete raw and processed proteomics data, together with detailed acquisition parameters and analysis outputs, have been deposited to the ProteomeXchange Consortium via the iProX partner repository under accession PXD072605.
